# Study on the Sealing Performance of a Composite Plugging System Comprising Cement and Sn58Bi Alloy for Wellbore Applications

**DOI:** 10.3390/ma18102301

**Published:** 2025-05-15

**Authors:** Chunqing Zha, Zhengyang Zhang, Wei Wang, Gonghui Liu, Jun Li, Wei Liu

**Affiliations:** 1 College of Mechanical and Energy Engineering, Beijing University of Technology, Beijing 100124, China; chachunqing@bjut.edu.cn (C.Z.); zhangzhengyang@emails.bjut.edu.cn (Z.Z.); lgh_1029@163.com (G.L.); 2 School of Engineering, China University of Petroleum (Beijing) at Karamay, Karamay 834000, China; lijun446@vip.163.com; 3 CNPC Engineering Technology R&D Company Limited, Beijing 102206, China; liuweidri@cnpc.com.cn

**Keywords:** CCUS, Sn58Bi alloy, composite plugging, sealing performance

## Abstract

To address the issue of sealing failure of cement materials commonly used as wellbore plugging agents in CO_2_ geological storage, this study proposes a composite wellbore plugging method that combines cement and Sn58Bi alloy. In this method, a composite sealing structure of “cement–Sn58Bi alloy–cement” is constructed within the wellbore. To evaluate the performance of this method, a series of pressure-bearing and gas-tightness experimental devices were designed, and experiments were conducted to assess the pressure-bearing capacity and gas sealing performance of the composite plugs. Additionally, optical microscopy was employed to observe and analyze the microstructure of the plugs. The effects of alloy proportion and temperature on the sealing performance of the composite plugs were systematically investigated. The experimental results indicate that both the pressure-bearing capacity and gas-tightness performance of the plugs are influenced by the alloy content and ambient temperature. Specifically, when the temperature increased from 30 °C to 60 °C, the pressure-bearing capacity decreased by an average of 28.3%; when further increased from 60 °C to 90 °C, it decreased by an average of 21.1%. In contrast, the gas-tightness performance exhibited an opposite trend, with the breakthrough pressure increasing by an average of 25.7% and 22.0%, respectively, over the same temperature intervals. Moreover, increasing the alloy proportion in the composite plugs enhanced both their pressure-bearing and gas-tightness performances. This study provides theoretical support for the application of composite plugs in CO_2_ geological storage.

## 1. Introduction

In recent years, the issue of global climate warming has intensified, and the international focus on carbon neutrality targets has increased significantly. Carbon Capture, Utilization and Storage (CCUS) technology is considered as a crucial pathway to achieving carbon neutrality [1]. Among the CCUS processes, geological storage of carbon dioxide, as a core component, aims to securely store CO_2_ in geological reservoirs over the long term, thereby preventing its migration to the surface and effectively reducing greenhouse gas emissions [2]. In this process, the performance of wellbore sealing materials is critical to ensuring the safety and effectiveness of geological storage. Once seal failure occurs, it may lead to CO_2_ leakage, environmental contamination, and even well blowout accidents, posing significant safety risks [3].

At present, cement materials are widely used in wellbore plugging due to their sealing capability, ease of construction, and environmental friendliness. In addition, cement plugs can bond closely with the wellbore wall and exhibit favorable load-bearing performance under mechanical stress, making Portland cement one of the most commonly used sealing materials. The primary component of Portland cement is calcium oxide (CaO), and its slurry typically has a pH value ranging from 13 to 14. However, studies by Huang et al. have shown that in CO_2_-rich geological reservoirs, carbon dioxide can react with cement through a neutralization process to form carbonate compounds, resulting in chemical corrosion of the cement sheath [4,5,6]. Further research by Quan et al. revealed that this corrosion not only weakens the mechanical properties of the cement but also reduces its long-term sealing capacity, becoming a major factor affecting the safety and reliability of underground CO_2_ storage operations [7].

To address the limitations of conventional cement-based sealing materials, researchers have explored various alternative materials, among which Sn58Bi alloy has attracted considerable attention due to its unique properties [8]. Compared with traditional materials, Sn58Bi alloy offers several significant advantages, including high density, low melting point, and excellent resistance to the corrosion of CO_2_ and H_2_S [9].

Defects in the sealing process using either cement plugs or Sn58Bi alloy plugs primarily arise from the inherent limitations of each material. To address the shortcomings of single-material sealing, this study proposes a dual-material composite plugging method that combines cement with Sn58Bi alloy. Specifically, this method leverages the excellent CO_2_ corrosion resistance of Sn58Bi alloy and the superior interfacial bonding capability of cement to enhance the overall sealing performance. The two materials function complementarily, effectively overcoming the application limitations associated with single-material plugs under complex downhole conditions.

Currently, this composite plugging method is still at a preliminary stage, with its practical application requiring further experimental validation and optimization. In this study, based on the material characteristics of Sn58Bi alloy and the operational demands of downhole plugging, two experiments were designed to evaluate sealing performance. A comparative analysis was conducted on the sealing effectiveness of composite plugs, single-cement plugs, and single-alloy plugs. The results provide experimental evidence supporting the feasibility and advantages of the composite plugging method and lay a theoretical foundation for its application in challenging downhole environments.

## 2. Properties of Sn58Bi Alloy and the Principle of Downhole Composite Plugging

### 2.1. Properties of Sn58Bi Alloy

Sn58Bi is a type of Bi-Sn alloy, consisting of 58% bismuth and 42% tin. Its properties are presented in Table 1.

When used as a plugging material, the high density of Sn58Bi alloy enables it to effectively expel internal impurities during solidification, forming a dense and uniform sealing layer. Its low melting point ensures that it can be melted in downhole environments without causing damage to the casing. Additionally, its excellent corrosion resistance effectively overcomes the limitations of cement-based materials in acidic gas environments such as CO_2_ and H_2_S [11]. Furthermore, as a eutectic alloy, Sn58Bi can bypass the gel phase during solidification, transitioning directly from liquid to solid phase, which significantly improves the plugging efficiency and stability.

**Figure 1:**
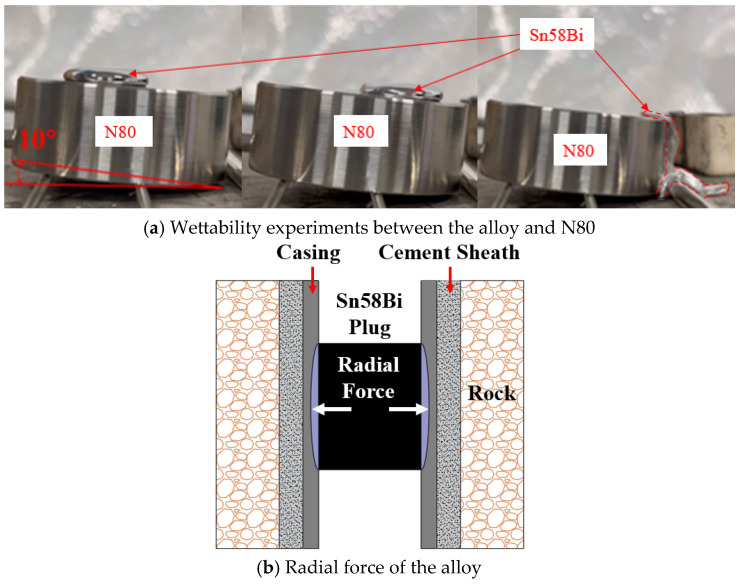
Wettability and expansion behavior of Sn58Bi alloy.

However, in its liquid state, Sn58Bi alloy does not effectively wet or adhere to the surface of N80 (a material that has been widely utilized as the primary casing material in oil well operations) steel plates; instead, it flows along the metal surface without bonding [12] (see Figure 1a). The intimate contact between the alloy and the casing primarily relies on the volumetric expansion effect that occurs during solidification. As the alloy solidifies, the radial force generated by volumetric expansion acts on the inner wall of the casing, enhancing surface friction and achieving mechanical sealing [13] (see Figure 1b). In view of this, to fully exploit the advantages of Sn58Bi alloy in downhole plugging applications and meet the demands of complex operational conditions, it is necessary to combine it with other materials.

### 2.2. Composite Plugging Method Using Sn58Bi Alloy and Cement in Wellbores

**Figure 2:**
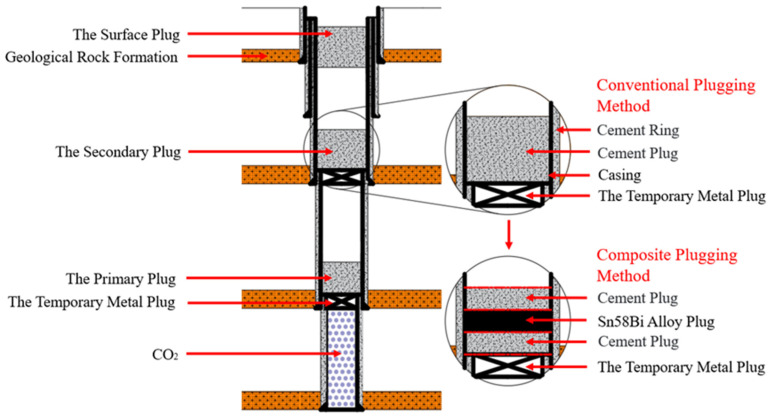
Schematic illustration of the downhole composite sealing method structure.

When cement is used as the plugging material, the cement plug is susceptible to corrosion by downhole CO_2_ and H_2_S gases. Conversely, when Sn58Bi alloy is applied as the sealing material, its lack of wettability prevents it from forming intimate contact with the wellbore wall; instead, it relies solely on volumetric expansion during solidification to achieve mechanical sealing, which necessitates combination with other materials. To overcome the respective limitations of these two sealing methods, a composite plugging method integrating both materials is proposed. Specifically, a multi-layered sealing structure of “cement–Sn58Bi alloy–cement” is constructed within the wellbore, as illustrated in Figure 2.

The composite plugging method is a downhole sealing method based on a multi-layer structural design. Its process involves the following steps: First, a layer of cement is placed within the wellbore. Subsequently, a heating rod equipped with an external Sn58Bi alloy shell is inserted into the well. Upon heating, the Sn58Bi alloy melts and bonds with the surrounding cement layer. After cooling, a dense alloy layer is formed, followed by the placement of an additional outer cement layer, thereby constructing a complete multi-layer sealing structure. This method potentially offers several advantages:

1. Enhanced sealing performance of Sn58Bi alloy: During solidification, the molten Sn58Bi alloy is susceptible to interference from rising gases in the wellbore, which may lead to the formation of internal channels that compromise the seal. The underlying cement layer acts as a protective barrier, preventing gas intrusion and ensuring uniform alloy solidification.
2. Improved CO_2_ corrosion resistance: The Sn58Bi alloy layer, with its excellent resistance to CO_2_ corrosion, can effectively protect the surrounding cement layers from chemical degradation, thereby enhancing the durability of the sealing structure.
3. Optimized mechanical sealing performance: The cement layer can achieve close bonding with the casing wall, compensating for the poor wettability of Sn58Bi alloy and its inability to directly adhere to the casing. This significantly improves the overall mechanical sealing performance of the composite structure.

Despite the potential advantages that the composite plugging method may possess, it currently remains at the incipient research stage. Compared with single-material plugging methods, its performance lacks sufficient experimental validation. This study investigates the mechanical sealing and gas-tight performance of the composite plug, providing a basis for future technical optimization and practical field applications.

## 3. Experimental Device

### 3.1. Preparation of Plug in Casing

**Figure 3:**
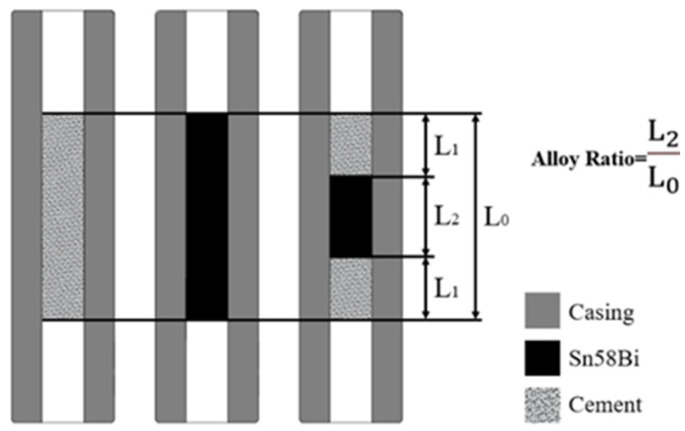
Cross-sectional morphology of the experimental sample.

The cement used in this study was standard Grade G cement, supplied by Qingyun Kangjing Building Materials Co., Ltd. (Dezhou, China). The casing used in the experiments had a height of 220 mm, an inner diameter of 20 mm, and an outer diameter of 50 mm, and was made of N80 steel, supplied by Shanghai Yinfan Metal Group Co., Ltd. (Shanghai, China). Within the casing, composite plugs with a “cement–Sn58Bi alloy–cement” structure were prepared. The total thickness of each plug was L_0_ = 70 mm, with the cement layers having thicknesses of L_1_ = 20 mm, 15 mm, and 10 mm, and the alloy layers having thicknesses of L_2_ = 30 mm, 40 mm, and 50 mm. Three plugs were prepared for each combination, yielding a total of nine composite plugs. Additionally, three pure cement plugs (70 mm thickness) and three pure Sn58Bi alloy plugs (70 mm thickness) were fabricated, resulting in six single-material plugs. The cross-sectional view of the experimental sample is shown in Figure 3, and the specific thickness of the plug is listed in Table 2.

### 3.2. Device for Mechanical Push-Out Experiment

**Figure 4:**
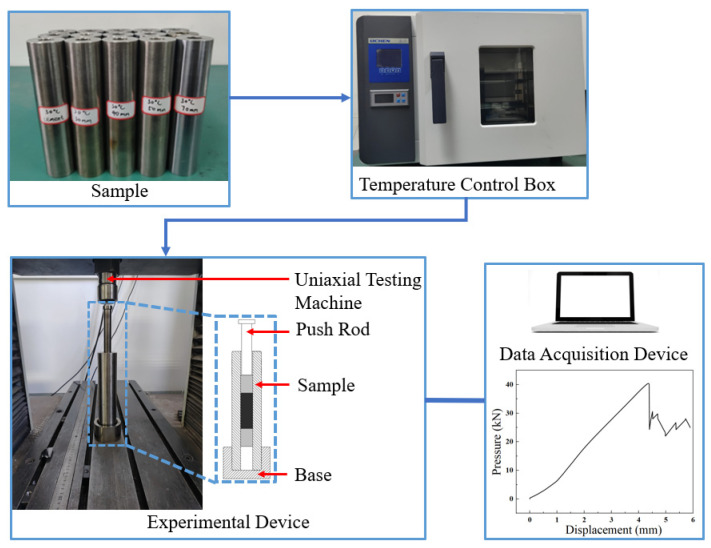
Schematic diagram of the mechanical push-out experiment.

When the external force applied to the plug exceeds its bearing capacity, axial slippage occurs between the plug and the casing, resulting in mechanical sealing failure. This failure can lead to CO_2_ leakage and subsequent environmental pollution. The mechanical sealing performance of different plugs was tested and compared by applying axial force using a uniaxial testing machine. When the axial thrust applied to the plug exceeded its bearing capacity, relative displacement occurred between the plug and the casing, which was considered indicative of mechanical sealing failure. At this point, the thrust recorded by the testing machine represented the maximum axial force that the plug could withstand. The schematic of the experimental equipment is presented in Figure 4.

### 3.3. Device for Gas Sealing Performance Experiment

**Figure 5:**
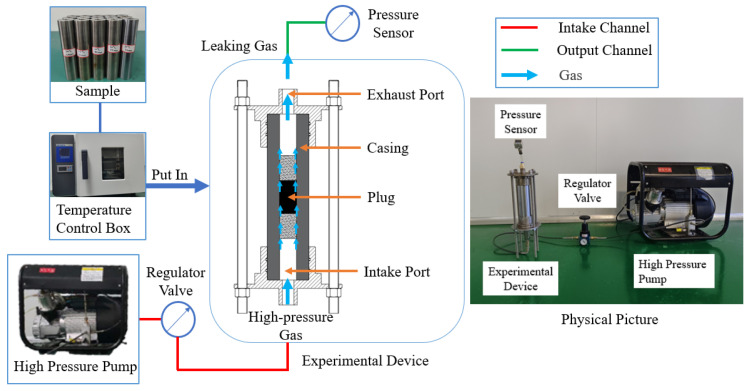
Connection diagram of gas sealing performance experimental device.

Gas sealing performance is one of the key indicators for evaluating the sealing effectiveness of plugs. When the downhole gas pressure exceeds the breakthrough pressure of the plug, continuous gas leakage channels may form within the plug, resulting in gas sealing failure and increasing the risk of gas escape. The gas sealing experiment apparatus is shown in Figure 5, consisting primarily of a high-pressure gas pump and a casing fixation and support device. The casing is sealed at both ends with upper and lower caps. One end serves as the gas inlet, connected to the high-pressure gas pump, with the input pressure controlled by a pressure regulating valve. The other end is connected to a gas pressure sensor to monitor any occurrence of gas leakage. When the applied gas pressure surpasses the breakthrough pressure of the sealing layer, a significant change in the sensor reading is observed, indicating gas sealing failure. The maximum gas pressure sustained by the plug is thereby determined [14,15].

## 4. Study on Performance of Composite Plug

### 4.1. Mechanical Push-Out Experiment

The temperature control chamber was adjusted to the required temperatures of 30 °C, 60 °C, and 90 °C and maintained for 30 min. The casing specimens were then placed inside the chamber, heated to the target experimental temperature, and held for 3 h. After heating, the casing was promptly removed and positioned on the uniaxial testing machine. The actuator rod of the testing machine was lowered to contact the plug, and the displacement was reset to zero. The pushing speed of the actuator was set to 3 mm/min, and the pressure–displacement curve was monitored in real time. The experiment was terminated once the curve exhibited a sudden drop, indicating the transition from static to dynamic friction and the occurrence of plug slippage. The actuator was then retracted, the casing removed, and the experimental data recorded and results summarized.

### 4.2. Gas Sealing Performance Experiment

The temperature settings of the temperature control chamber were maintained consistent with those used in the push-out experiments. After heating, the casing specimens were removed from the chamber and mounted onto the gas sealing experiment apparatus. The casing was secured and tightened using upper and lower end caps, with double sealing rings applied between the casing and the caps to ensure sealing integrity. A pressure sensor was connected to the upper end cap, while the lower end cap was connected to a high-pressure gas pump controlled by a pressure regulating valve.

Prior to the experiment, sealing effectiveness was verified by initiating the gas pump and adjusting the regulating valve to supply gas briefly. The pressure sensor readings were monitored; if the readings remained stable or exhibited minimal fluctuations, effective sealing was confirmed. Subsequently, the inlet gas pressure was gradually increased until a significant change in the pressure sensor reading was observed, indicating plug failure. At this point, pressurization was halted, and the breakthrough pressure was recorded once the data stabilized. Upon completion of the experiment, all experimental data were recorded and the results were summarized and analyzed.

## 5. Experimental Analysis

### 5.1. Mechanical Push-Out Experiment Analysis

**Figure 6:**
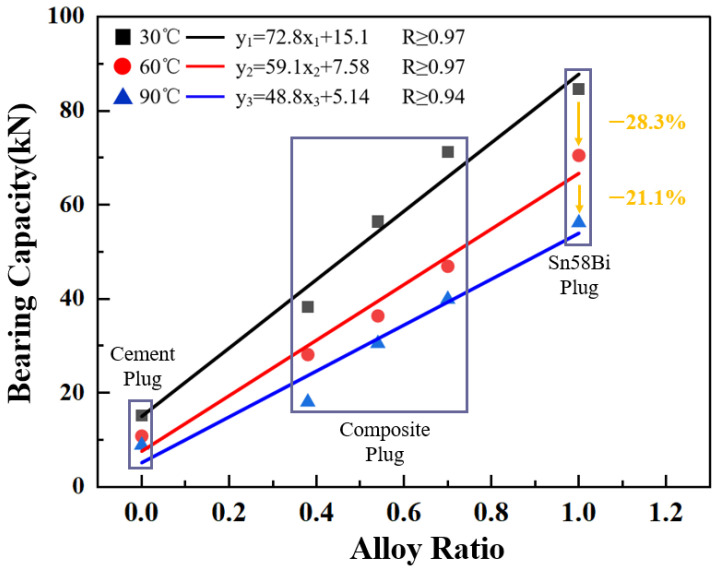
Influence of ambient temperature and alloy ratio on the bearing capacity of plugs.

As the ambient temperature increases, Sn58Bi alloy exhibits creep behavior, which can subsequently alter its mechanical properties, potentially affecting its sealing performance. To investigate the influence of environmental temperature on the bearing capacity of the plugging assembly, this study conducted a series of experiments to obtain the pressure-bearing performance data of composite plugs with three different alloy-to-cement ratios under various temperature conditions. The results were then compared with those of pure cement plugs and pure Sn58Bi alloy plugs. The experimental results are presented in Figure 6.

The experimental results indicate the following:

1. With increasing ambient temperature, the bearing capacity of the plugging assemblies exhibited a significant decreasing trend. When the temperature rose from 30 °C to 60 °C, the average bearing capacity of the plugs decreased by approximately 28.3%. A further increase to 90 °C resulted in an additional average reduction of about 21.1% compared to that at 60 °C. It is noteworthy that the reduction in bearing capacity for cement plugs at different temperatures was consistently lower than the average decline, suggesting that the bearing capacity of Sn58Bi alloy plugs is more sensitive to temperature variations than that of cement plugs.
2. Elevated ambient temperature markedly weakened the bearing capacity of the plugging assemblies, with the rate of reduction becoming more pronounced beyond 60 °C. This observation indicates a notable deterioration in the sealing performance of the plugging system under high-temperature conditions, which can be attributed to the enhanced creep behavior of the Sn58Bi alloy at elevated temperatures [16], resulting in a significant degradation of the mechanical performance of both the composite plugging layers and the pure Sn58Bi alloy plugs.
3. Under constant temperature conditions, the bearing capacity of the plugs increased progressively with higher alloy content. Across all three experimental groups, both the composite plugs and the pure Sn58Bi alloy plugs demonstrated superior bearing performance compared to pure cement plugs, confirming that the Sn58Bi alloy material exhibits better mechanical sealing performance and pressure-bearing capability than cement in downhole applications.

The results of this experiment are similar to those reported by Liang Bin et al. [17], demonstrating that the mechanical properties of Sn58Bi alloy degrade at elevated temperatures. Consistent findings were also observed by Pang et al. [18], who reported an approximately linear relationship between temperature and mechanical performance.

### 5.2. Gas Sealing Performance Experiment Analysis

**Figure 7:**
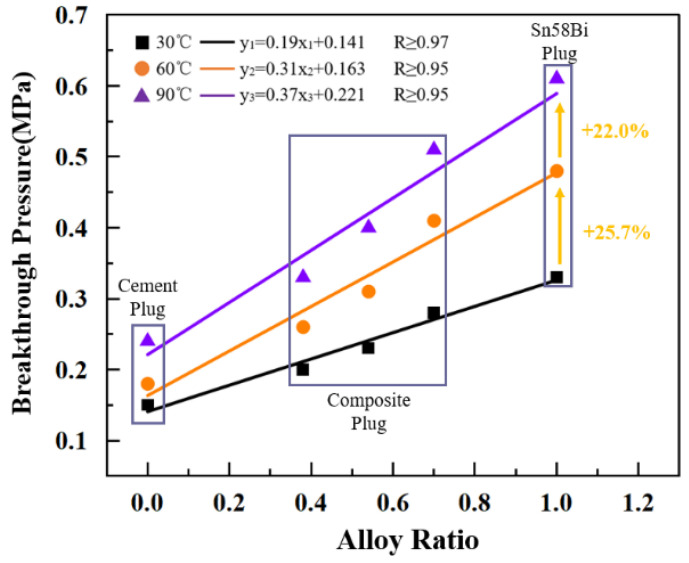
Influence of ambient temperature and alloy ratio on the breakthrough pressure of plugs.

Due to the differences in the coefficients of thermal expansion among cement, Sn58Bi alloy, and casing materials, volumetric expansion occurs in the specimens during heating, which may subsequently affect the gas-tight sealing performance of the plugs. To further investigate the influence of ambient temperature on the gas sealing capacity of the plugs, a series of experiments were conducted. The gas sealing performance of composite plugs with three different alloy proportions was evaluated under various temperature conditions, and the results were compared with those of pure cement plugs and pure Sn58Bi alloy plugs. The experimental results are presented in Figure 7.

The experimental results indicate the following:

1. With increasing ambient temperature, the breakthrough pressure of the plugs exhibited a significant upward trend. When the temperature increased from 30 °C to 60 °C, the average breakthrough pressure rose by approximately 25.7%; further increasing the temperature to 90 °C resulted in an additional average increase of about 22.0% compared to 60 °C. It is noteworthy that the variation in breakthrough pressure for the cement plug across different temperature conditions was relatively small, indicating that the gas-tight sealing performance of Sn58Bi alloy plugs is more sensitive to temperature variations than that of cement plugs.
2. The rise in ambient temperature contributed to an improvement in the gas sealing performance of the plugs. This enhancement is likely attributed to the mismatch in thermal expansion coefficients between the sealing materials and the casing material. As the plugs undergo thermal expansion during heating, they achieve tighter contact with the inner wall of the casing, thereby effectively reducing the risk of gas leakage and enhancing sealing integrity. This trend persisted even as the temperature increased from 60 °C to 90 °C.
3. Under isothermal conditions, increasing the proportion of Sn58Bi alloy in the plugs significantly enhanced their gas sealing performance. The results of all three groups of experiments demonstrated that both the composite plugs and the pure Sn58Bi alloy plugs exhibited superior gas-tight sealing capacities compared to pure cement plugs. This improvement can be attributed to the higher thermal expansion coefficient of Sn58Bi alloy relative to cement, resulting in more pronounced volumetric expansion under identical temperature variations. Consequently, a greater radial force was generated, leading to a tighter fit between the plug and the casing, thereby further improving the gas sealing performance of the plugging system.

The experimental results are consistent with those reported by Cui et al., indicating that the sealing performance of the Sn58Bi alloy is significantly affected by temperature. As the ambient temperature increases, the Sn58Bi alloy conforms more closely to the casing, resulting in improved sealing performance for both the alloy plug and the composite plug [19].

To ensure the long-term safety of CO_2_ storage, the CO_2_ is typically transported and injected in a supercritical state, which requires a minimum pressure of 7.38 MPa and a critical temperature of 31.1 °C. Under a geothermal gradient of 30 °C/km and a surface temperature of 10 °C, the minimum depth required to maintain supercritical conditions is approximately 800 m [20]. Assuming an injection tubing inner diameter of 139.7 mm, and in accordance with the recommendations of the API (American Petroleum Institute), the length of the cement plug should generally exceed 30 m, yielding a length-to-diameter ratio greater than 200. Experimental results demonstrate that, under identical ambient temperature conditions, the composite plugs exhibit better sealing performance than cement plugs with the same length-to-diameter ratio. They possess reliable sealing integrity and are capable of meeting the sealing requirements for CO_2_ storage.

### 5.3. Optical Microscopy Observation of Plugging Interface

**Figure 8:**
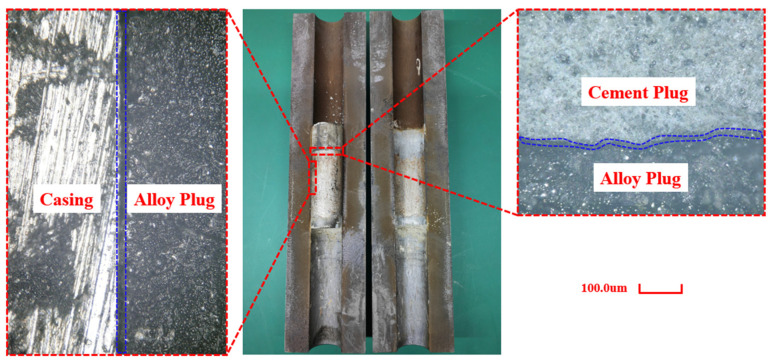
Optical microscopy of the casing/combined plug interface.

After the experiment, the combined plug formed at 90 °C with an internal alloy layer thickness of 40 mm was cut open. The interfaces between the casing/Sn58Bi alloy plug and the cement plug/Sn58Bi alloy plug were observed under a microscope, as shown in Figure 8. It was found that gaps existed at both the casing/Sn58Bi alloy plug interface and the cement plug/Sn58Bi alloy plug interface. This phenomenon may be attributed to the differences in thermal expansion characteristics between the different materials.

## 6. Conclusions

In this study, the mechanical and gas sealing performance of a composite wellbore plugging method utilizing cement and Sn58Bi alloy was systematically investigated. The sealing performance of the composite plug was compared with that of single-material plugs under varying temperature conditions. The following conclusions were drawn:

1. As a novel downhole casing sealing material, Sn58Bi alloy, when used in combination with cement to form a composite plug, demonstrated superior mechanical and gas sealing performance compared to a pure cement plug, and slightly inferior performance to a pure Sn58Bi alloy plug.
2. In terms of mechanical sealing performance, the bearing capacity of all plug types decreased with increasing temperature. The reduction in bearing capacity was positively correlated with the proportion of alloy within the plug. Additionally, the presence of cement at both ends of the composite plug mitigated the extent of mechanical performance degradation under high-temperature conditions compared to the pure Sn58Bi alloy plug.
3. With regard to gas sealing performance, the gas-tightness of the plugging systems improved with increasing temperature, and the degree of improvement was likewise positively correlated with the proportion of Sn58Bi alloy. This enhancement is attributed to the significant thermal expansion of the Sn58Bi alloy at elevated temperatures, which improves its conformity to the casing wall, thereby enhancing the gas sealing effectiveness.
4. Increasing the proportion of Sn58Bi alloy within the plug contributed to simultaneous improvements in both the mechanical and gas sealing performances. Furthermore, experimental results revealed that the bearing capacity and breakthrough pressure of the composite plug exceeded the theoretical linear sum of those for the pure alloy and pure cement plugs, indicating a synergistic advantage in sealing performance achieved through the composite design.

## Figures and Tables

**Table 1:** The properties of Sn58Bi alloy [10].

Density /g × cm^−3^	Melting Point/°C	Volume Change (Liquid to Solid)	Elastic Modulus /GPa	Tensile Strength /MPa	Coefficient of Thermal Expansion/°C^−1^
8.72	138	+0.77%	47.2	71.7	1.5 × 10^−7^

**Table 2:** Plug size specifications.

Plug	L_1_/mm	L_2_/mm	L_3_/mm
pure cement plug	0	70	0
pure Sn58Bi alloy plug	0	70	0
composite plug	20	30	20
	15	40	15
	10	50	10

## Data Availability

The original contributions presented in the study are included in the article; further inquiries can be directed to the corresponding author.
